# The impact of inadequate prenatal care on maternal anemia and pregnancy outcomes in Romania

**DOI:** 10.25122/jml-2025-0074

**Published:** 2025-04

**Authors:** Giorgia Zampieri, Cringu Antoniu Ionescu

**Affiliations:** 1 Faculty of Medicine, University of Medicine and Pharmacy Carol Davila, Bucharest, Romania; 2 Department of Obstetrics and Gynecology, Sf. Pantelimon Emergency Clinical Hospital, Bucharest, Romania

**Keywords:** prenatal care, antenatal care, high-risk pregnancy, anemia, iron deficiency, blood transfusion

## Abstract

Prenatal care aims to ensure the well-being of both mother and baby by closely watching for potential problems and addressing them promptly. A lack of such care often stems from a combination of issues, including financial hardship, limited education, challenges in accessing healthcare, inadequate medical services, and insufficient social support. The study aimed to assess how iron supplements given during prenatal care affect the prevalence of anemia and whether lack of prenatal care leads to increased maternal and fetal morbidity. This analysis is based on the retrospective evaluation of medical data from 125 patients admitted to the Obstetrics and Gynecology Department of Sf. Pantelimon Hospital in Bucharest, Romania, from August 2024 to February 2025. The parameters considered in this study include prenatal care access, mode of delivery, antepartum anemia and its severity, iron supplementation, postpartum anemia and the necessity of blood transfusions, and length of hospitalization. The link between prenatal care and reduced antepartum anemia underscores the importance of monitoring during pregnancy to mitigate risks. Postpartum hemoglobin levels were significantly higher in patients who received prenatal care, leading to a decrease in severe and moderate anemia cases. Additionally, prenatal care was associated with shorter hospital stays for newborns. Comprehensive prenatal care demonstrably enhanced both maternal and neonatal prognoses across the continuum of pregnancy and the puerperium, thereby contributing to a significant reduction in rates of maternal and fetal morbidity and mortality.

## INTRODUCTION

Prenatal visits promote maternal and fetal health by carefully monitoring risk factors and treatment of complications as soon as they arise [1]. Clinical and psychological outcomes during pregnancy and the postpartum period are improved by proper prenatal care, thus reducing maternal and fetal morbidity and mortality [2]. Lack of prenatal care refers to the insufficient or complete absence of medical care and support provided to a pregnant woman during her pregnancy [1]. The main pathologies observed that are associated with the lack of prenatal visits are premature birth, intrauterine growth restriction, or low birth weight, as well as the occurrence of anemia and maternal or fetal infections in the prenatal or postnatal period [1,3,4].

It has been demonstrated that the lack of prenatal care is mainly the result of the negative impact of several factors, including socioeconomic factors, such as low income and lack of education, difficult access to health services, the quality of medical services, and lack of social support [1,5,6]. Over time, certain categories of patients have been identified as more likely to attend fewer prenatal visits and, in some cases, receive no prenatal care until delivery [7–9]. Financial barriers, particularly the pregnant woman's economic situation, play a major role in limiting access to care [2,10,11]. It has been shown that a precarious financial situation is extremely detrimental to the degree of prenatal care, which is why free health services during pregnancy have been tried to remedy this problem. Unfortunately, other frequently encountered risk factors lead to the absence of prenatal visits, such as lack of education in the population [1,2,7].

A low level of education has been strongly associated with the absence of prenatal care, making it one of the main reasons for limited access to medical assistance in general [1,2]. In contrast, higher levels of education have been linked to better pregnancy monitoring, even among women from lower socioeconomic backgrounds [10,12]. Additionally, various studies have shown that pregnant women without a partner tend to have fewer prenatal visits compared to those who are married, as partner support during pregnancy encourages adherence to prenatal care programs [3]. In contrast, the lack of support from the partner contributes to the lack of prenatal care, with a reduced number of consultations during pregnancy [1,3].

Multiparity has been demonstrated to be a risk factor for the lack of prenatal care compared to primiparity [5,6,11]. Studies have shown that multiparous women, especially those without a history of obstetric complications, are less likely to seek medical services during pregnancy, especially those who do not have family support [7]. Furthermore, a short interval between two pregnancies has also been associated with reduced utilization of prenatal care services [7,10,13]. It has been shown that patients who have had unpleasant experiences with the hospital environment— whether during prenatal visits, childbirth, or the postpartum period—often develop increased resistance to prenatal care recommendations and frequently reject the idea of attending prenatal consultations [12]. To address this issue and rebuild trust in the medical system, several measures should be considered to create a more favorable environment, such as respect for patients, for their questions, time, and problems, increasing accessibility through a predictable environment, flexibility, responsiveness, and overall patient-centered care [7,12].

Another group at increased risk of inadequate prenatal care includes young women, particularly adolescents. Similarly, advanced maternal age, particularly in women over 40 years, has also been linked to lower rates of prenatal care utilization [6,14-16].

Additionally, it has been shown that pregnant patients who use tobacco, alcohol, or illegal substances are at increased risk of receiving poor prenatal care [2,17]. Unplanned pregnancies are also directly associated with reduced utilization of medical services during pregnancy if the pregnancy is continued [1,12,18]. It is important to note that a significant proportion of maternal deaths are caused by complications related to abortion [18-20]. Unwanted pregnancies can be associated with unsafe abortion techniques, which can lead to increased maternal mortality, and the prevention of unwanted pregnancies is a critical point in health systems [19,21-23]. Psychological factors, such as denial of pregnancy or depressive syndromes, are also major reasons why some patients avoid prenatal visits [21,22]. Access to prenatal services, however, depends not only on the availability, quality of care, facilities, and financial resources but also on the woman’s perception of the need for prenatal visits and whether she considers them useful and acceptable [5,11,23]. Additionally, cultural and religious values also impact this decision [24].

In contrast, multiple studies have identified patient groups more likely to receive appropriate and comprehensive prenatal care. These include women with multiple pregnancies, comorbid conditions such as diabetes and hypertension, a history of antepartum vaginal bleeding, or diagnosed anxiety disorders [2,3,5,25]. In some cases, an excess of prenatal visits can even be observed, with no real benefit on the outcome of the pregnancy, but with excessive and improper use of financial resources [4].

Anemia in pregnancy is a significant public health concern impacting women around the world, with prevalence rates differing across regions [26]. This variation is significantly influenced by economic status, healthcare access, nutritional deficiencies (particularly iron and folic acid), and comorbidities [26,27]. The World Health Organization (WHO) estimates that around 37% of pregnant women globally are affected by anemia, with particularly high rates noted in low- and middle-income countries [28]. Recent statistics indicate that the prevalence of gestational anemia in Romania is 25.3% [29,30].

Maternal anemia is linked to a heightened risk of negative pregnancy outcomes [30]. During pregnancy, anemia can compromise maternal health, contributing to complications such as premature rupture of membranes and postpartum hemorrhage. In addition, it negatively impacts fetal development, raising the likelihood of low birth weight, preterm delivery, and neonatal asphyxia [30]. The mechanisms behind these outcomes are complex and biologically plausible. Anemia reduces the oxygen-carrying capacity of maternal blood, prompting compensatory adjustments within the body [31]. For example, the body increases cardiovascular and peripheral vascular pressure to satisfy the oxygen requirements of the fetus, which can lead to gestational hypertension [32]. Anemia reduces hemoglobin levels in maternal blood, diminishing its oxygen-carrying capacity and limiting the fetus’s access to adequate oxygen, which could result in hypoxia and asphyxia [30,33,34]. Postpartum hemorrhage is a major cause of mortality, and several studies have shown that severe anemia during pregnancy elevates the risk of postpartum hemorrhage, which may be linked to increased nitric oxide secretion and decreased contractility of uterine smooth muscles [35,36].

Postpartum anemia is defined as a decrease in hemoglobin levels below 11 g/dl within the first 42 days following delivery [37]. In developing countries, the prevalence of postpartum anemia ranges from 50% to 80% [37,38]. Research indicates that women who were anemic before delivery are about four times more likely to experience postpartum anemia than those who were not anemic [37,39]. Maternal health issues associated with postpartum anemia include depression, fatigue, and cognitive impairment, which can lead to decreased physical and mental performance, potentially affecting the bonding between mother and child and reducing the duration of breastfeeding [40-42].

The research gap highlights the need to consider diverse populations and geographical variability. This study specifically focuses on Romania, where access to prenatal care may be limited in certain regions.

## MATERIAL AND METHODS

The fundamental objective of prenatal care is to optimize the health status of pregnant women and the fetus by carefully monitoring risk factors and providing early therapeutic intervention in the event of complications. Adequate pregnancy monitoring significantly improves clinical and psychological outcomes both during the gestation and postpartum periods.

The hypothesis of this study was based on the suspicion that antenatal care reduces maternal and fetal morbidity by decreasing the number of cases of anemia during pregnancy and postpartum and by reducing the number of days of hospitalization. In order to rigorously validate the formulated working hypothesis, the following specific objectives were established, each aimed at contributing to a comprehensive understanding of the investigated phenomena and providing a solid foundation for the conclusions of the study:

- Detailed identification of the demographic, social, and medical characteristics of the patients. This objective aimed to create a comprehensive profile of the study participants through a thorough medical history and a complete clinical examination. Collecting demographic data (age, ethnicity, marital status, education level), social information (occupation, income, access to medical services), and medical history (obstetric history, pre-existing conditions, medication treatments) is essential for identifying potential risk factors and evaluating their impact on the studied variables.
- Rigorous quantification of relevant variables. This objective involved the precise measurement and recording of a series of variables considered relevant to the study of antepartum and postpartum anemia. These variables included age, parity, presence of prenatal care, mode of delivery, presence of antepartum and/or postpartum anemia, administration of iron supplements during pregnancy, values of hemoglobin, hematocrit, mean corpuscular volume (MCV), mean corpuscular hemoglobin (MCH), mean corpuscular hemoglobin concentration (MCHC), platelet, white blood cells, and neutrophils, presence of infections diagnosed through bacteriological examination of vaginal discharge, quantification of postpartum hemoglobin, determination of the severity of postpartum anemia, blood transfusion requirements, quantification of the number of days of hospitalization, and antibiotic therapy needs among newborns.
- Identification of patients with anemia. This objective aimed to determine the prevalence of anemia among the study participants, both antepartum and postpartum. Accurate identification of patients with anemia is essential for assessing its impact on the course of pregnancy and perinatal outcomes.
- Identification of correlations between the lack of prenatal care and anemia. This objective aimed to investigate the relationship between the absence of adequate prenatal care and the risk of anemia. It is presumed that the lack of prenatal care may be associated with a higher likelihood of developing anemia due to the absence of monitoring of the patient's health status and the lack of preventive interventions, such as iron supplementation.
- Evaluation of how the phenomenon of lack of prenatal care influences newborns' hospitalization length. This objective aimed to investigate the impact of the lack of prenatal care on the health of newborns. It is presumed that the absence of prenatal care may be associated with an increased risk of neonatal complications, which can lead to a longer hospitalization period for newborns.

Medical data from 125 patients who delivered at the Obstetrics and Gynecology department of St. Pantelimon Emergency Clinical Hospital between August 1^st^, 2024, and February 28^th^, 2025, were retrospectively analyzed for this single-center study. The research plan was implemented in accordance with the norms of scientific, professional, and university ethics, in compliance with the provisions of the code of ethics and deontology of the involved institutions.

To establish the required database, the study included adult patients over 18 years old who consented to the processing of their medical data for scientific purposes upon admission, as well as minors over 16 years old whose legal guardians provided consent for the processing of medical data. Participants were required to have a single pregnancy with a gestational age between 37 and 41 weeks. The exclusion criteria comprised individuals under 16 years old, patients who declined to allow the processing of their medical data for research purposes at the time of admission, and those with multiple pregnancies or preterm births.

The foundation of this research involved combining the data found in the general medical observation sheet of the patients, the treatment plan, and the results of the blood tests.

The elements of descriptive statistics highlighted the following components:

- Quantitative variables: age (completed years), parity, hemoglobin, hematocrit, MCV, MCH, MCHC, platelets, white blood cells, neutrophils, postpartum Hb, number of hospital days for the mother, number of hospital days for the newborn, Apgar score;
- Dichotomous qualitative variables: prenatal care (yes/no), mode of delivery (vaginal/c-section), antepartum anemia (yes/no), iron supplements antenatally (yes/no), iron supplements postpartum (yes/no), blood transfusion (yes/no), need for antibiotics for the newborn (yes/no);
- Nominal qualitative medical variables: severity of anemia (mild/moderate/severe), cervical swab (positive/negative/not tested).

Following data collection, an Excel database was created for statistical analysis. IBM SPSS Statistics for Windows, Version 29.0 (30-day trial version), Armonk, NY: IBM Corp was used to process the data. Categorical variables are presented as frequencies and percentages, while continuous variables are presented with descriptive statistics (mean, standard deviation, median, minimum, and maximum). Associations between categorical variables were assessed using chi-square tests or Fisher's exact test when chi-square assumptions were not met. Pearson's correlation coefficient was calculated to determine the degree of correlation between parameters. Independent samples *t*-tests were used to compare means for dichotomous variables, and ANOVA was used to compare means between multiple groups. Statistical significance was defined as *P* < 0.05.

## RESULTS

**Figure 1:**
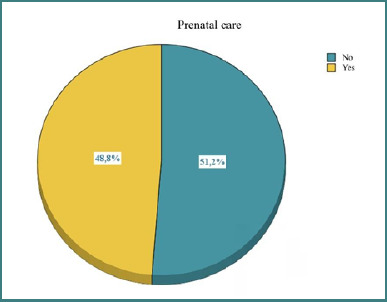
Distribution of patients based on access to prenatal care

The first part presented in the results section is represented by the descriptive statistical analysis. Regarding the number of patients who received prenatal care, the proportions are relatively balanced, but slightly more people (51.2%) did not receive medical care during pregnancy. The patients’ distribution is illustrated in Figure 1.

Regarding the number of previous births, among the 125 patients included in the study, most patients (44%) were primiparous. The group was predominantly composed of young individuals, with a concentration around 19-25 years old. The youngest mother was 16 years old, and the oldest was 40 years old, which reflects a relatively wide age range.

### Correlations between prenatal care, parity, and maternal age

**Figure 2:**
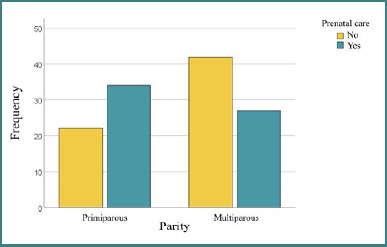
Correlations between parity and access to prenatal care

The statistical analysis revealed a significant association between age and prenatal care. Medical care appears to be more common among older individuals, reflecting an increased need for monitoring with advancing age or improved access to healthcare services. First-time mothers (primiparous women) were more likely to be registered for antenatal care, whereas women with multiple previous births (multiparous women) were more often unregistered, as shown in Figure 2. These findings suggest that primiparity may increase the likelihood of receiving prenatal care (*P* < 0,05). The results suggest a possible inequality in access to healthcare services for women with multiple children. This may be related to available resources, the prioritization of younger or smaller groups, or other social or medical barriers.

### Correlations between prenatal care and mode of delivery

Regarding the method of delivery, 38,4% of patients gave birth vaginally, while the majority (61,6%) underwent c-section. The high proportion of cesarean births suggests a preference or increased need for this method. Medical factors (complications, maternal or fetal risks), cultural factors, personal preferences, or policies of medical units may be involved.

**Figure 3:**
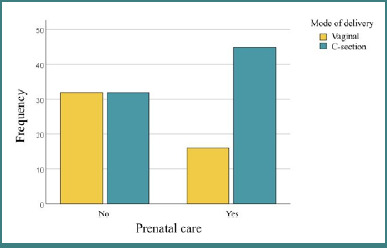
Correlations between mode of delivery and access to prenatal care

The results indicate a significant association between prenatal care and mode of delivery, with a higher proportion of c-sections among women receiving care (*P* = 0,006), as seen in Figure 3. This finding may be explained by the fact that patients who did not access healthcare services are often admitted to the hospital in advanced stages of labor.

### Correlations between prenatal care and antepartum anemia

**Figure 4:**
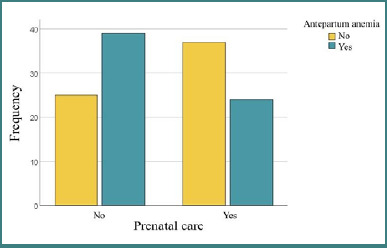
Correlations between prenatal care and antepartum anemia

Antepartum anemia affected a significant proportion of the studied group (50.4%), highlighting the need to monitor and manage this condition in prenatal care. Prenatal care was associated with a lower prevalence of antepartum anemia (*P* = 0.016), underscoring the importance of prenatal monitoring for reducing the risks associated with this condition. Women who did not access antenatal care had a higher prevalence of antepartum anemia (60.9%) compared to those who received medical care (39.3%), as illustrated in Figure 4. This may be related to improved access to iron supplements, nutritional counseling, or regular monitoring during pregnancy.

**Figure 5:**
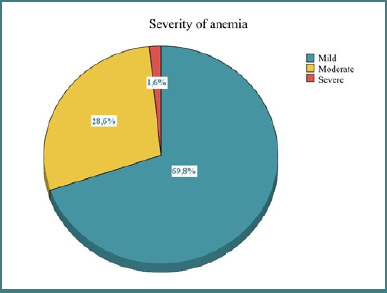
Distribution of patients based on the severity of anemia

Approximately 55.6% of antepartum anemia cases were mild (44 out of 63), as shown in Figure 5. Only one person (0.8%) had severe anemia, indicating a very low prevalence of severe anemia.

Statistical analysis did not reveal a significant association between prenatal care and the severity of antepartum anemia, as indicated by a *P* value greater than 0.05. However, women who received prenatal care generally presented with milder forms of anemia and no cases of severe anemia compared to those not receiving medical care, who had a more varied distribution, including a small percentage of severe cases. Iron administration appears to be associated with a slight reduction in severe anemia cases, with no severe cases in the group that received iron supplements.

### Correlations between prenatal care and cervical swab results

Regarding cervical swab results in the study population, most cases (67.2%) had negative cultures, suggesting a low prevalence of bacterial infections. However, 19.2% of cases lacked available results, which may limit the completeness of data interpretation. Nearly 14% of cases had positive cultures, indicating a need for monitoring and treatment for these positive cases to prevent possible complications.

Prenatal care status was significantly associated with cervical swab results at 36 weeks of gestation. Individuals who received prenatal care had a higher proportion of negative results (86.9%) and fewer positive results (13.1%) than those without prenatal care, who had 48.4% negative and 14.1% positive results. Additionally, there were no missing results among women who received prenatal care, suggesting more comprehensive monitoring in this group.

### Correlations between prenatal care and antepartum iron supplementation

**Figure 6:**
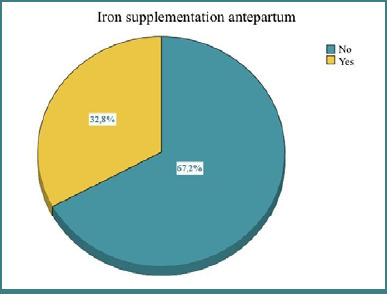
Distribution of patients based on iron administration

Approximately 67.2% of the women did not receive iron antepartum, while 32.8% benefited from iron administration. Nearly 90% of individuals who received iron benefited from healthcare services, while only 9.8% of mothers who received iron did not benefit from regular monitoring. In the group of mothers who received iron, 29.3% had mild anemia, and 14.6% had moderate anemia. The distribution observed in Figure 6 suggests that a significant proportion of women did not undergo iron treatment.

A significant correlation was observed between antepartum iron administration and prenatal care. Women who received antepartum iron were much more likely to access health care than those who did not. These results suggest a link between careful medical monitoring and iron administration during the antepartum period.

### Correlations between prenatal care and hematological parameters

In the study group, the average hemoglobin value was 11.1 g/dL, with a relatively small variation (the standard deviation was 1.32 g/dL). The values ranged from 6.5 to 14.4 g/dL, indicating the presence of both anemic cases (Hb < 11 g/dL) and individuals with normal hemoglobin values. Although most hematological parameters fell within normal limits, the wide range of values suggests the existence of atypical or extreme cases within the cohort.

Participants who accessed prenatal care had more favorable hematological parameters than those who did not, suggesting a better health status in the medically monitored group (Table 1). In contrast, the group without prenatal care showed a slightly lower average hemoglobin level, and the range indicated the presence of anemia in some cases. The standard deviation suggested moderate variability in this subgroup. Hematocrit (Hct) values also varied more in the non-prenatal care group, with the mean slightly below 33%. By comparison, antenatal care patients had a higher average Hb level and Hct values, indicating fewer cases of severe anemia. Furthermore, MCH values were slightly higher in the prenatal care group. The WBC was lower in the group who received prenatal care, indicating fewer infections or inflammations.

**Table 1:** Descriptive analysis of hematological parameters

Prenatal care		*n*		Mean	Median	Standard deviation	Min	Max
		Valid data	No data					
No	Hb	64	0	10,839	10,700	1,3735	6,5	14,4
	Hct	64	0	32,916	33,300	3,1743	22,8	41,3
	MCV	64	0	83,292	83,850	7,3268	61,0	98,7
	MCH	64	0	27,450	27,850	3,4391	17,4	34,4
	MCHC	64	0	33,508	32,950	5,0533	28,5	71,4
	PLT	64	0	272,41	271,00	61,272	134	472
	WBC	64	0	11,8916	10,9500	3,25827	6,50	22,98
	Neutrophils	64	0	8,6788	7,7550	3,00524	4,56	17,86
Yes	Hb	61	0	11,372	11,500	1,2054	8,8	13,3
	Hct	61	0	34,500	34,900	3,1001	28,3	41,0
	MCV	61	0	83,907	84,300	5,2156	67,3	93,8
	MCH	61	0	27,670	28,100	2,4632	20,5	31,6
	MCHC	61	0	32,928	32,900	1,3250	30,2	36,5
	PLT	61	0	252,15	249,00	62,199	112	408
	WBC	61	0	10,6061	9,9700	2,66565	6,21	21,08
	Neutrophils	61	0	7,5915	6,7600	2,30804	3,97	15,83

**Table 2:** Summary table

Prenatal care and maternal age	Medical care was more common among older individuals.
Prenatal care and parity	Primiparous women tended to access prenatal care more frequently.
Prenatal care and mode of delivery	Higher proportion of births by c-section among patients who received prenatal care.
Prenatal care and antepartum anemia	Prenatal care was associated with a lower prevalence of antepartum anemia.
Prenatal care and severity of antepartum anemia	No significant correlation.
Prenatal care and cervical swab results	Prenatal care significantly influenced the results of cervical swabs at 36 weeks.
Prenatal care and antepartum iron supplementation	There was a link between careful monitoring and iron administration.
Prenatal care and postpartum anemia	Postpartum Hb levels were significantly higher in patients who accessed prenatal care.
Prenatal care and postpartum need for transfusion	No significant correlation.
Mode of delivery and postpartum anemia	No significant correlation.
Prenatal care and length of hospitalization	Babies born to mothers who received medical care tended to have a shorter hospital stay.
Prenatal care and antibiotic administration in the newborn	Babies born to mothers who received prenatal care might be less likely to receive antibiotics.

### Correlations between prenatal care and postpartum anemia

**Figure 7:**
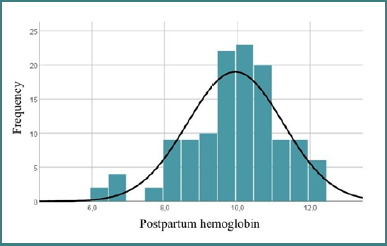
Distribution of patients based on postpartum hemoglobin

The average postpartum Hb of 9.942 g/dL and the minimum value of 6.2 g/dL could indicate a significant prevalence of anemia in the cohort. Most values were relatively close to the average, as indicated in Figure 7, but some patients exhibited very low values, indicating severe anemia.

There was a statistically significant difference between postpartum Hb levels in patients receiving prenatal care and those not attending medical visits. Patients who were monitored had higher postpartum Hb values. The average difference of almost 0.5 g/dL suggests a clinical benefit of monitoring during pregnancy. Prenatal care may contribute to the prevention of postpartum anemia.

### Correlations between prenatal care and postpartum need for blood transfusions

Regarding the necessity of blood transfusion, most patients did not require a postpartum transfusion. A small percentage (4%) of patients required a transfusion due to severe postpartum anemia. With a *P* value greater than the significance threshold, no statistically significant association between prenatal care and the need for transfusion was found. Although the differences between groups were observable, these were not strong enough to rule out the possibility of random variation.

### Correlations between the mode of delivery and postpartum anemia

No statistically significant difference was observed between the average postpartum Hb values of patients who delivered vaginally and those who underwent cesarean section. The results indicate that the mode of delivery did not significantly influence postpartum Hb levels. There was also no statistically significant association between the mode of delivery and the need for postpartum transfusion. As for iron supplementation, antepartum iron administration did not appear to directly influence the need for postpartum transfusion. However, the need for transfusion might be better explained by factors such as the severity of prenatal anemia, blood loss during delivery, or other obstetrical complications.

### Correlations between prenatal care and length of hospital stay

A significant difference was observed in the length of hospital stay for infants based on maternal prenatal care status (t = 2.737, *P* = 0.007). Infants born to mothers who received prenatal care had shorter hospital stays, which may reflect better overall health at birth. While antenatal care plays an important role in monitoring maternal and fetal health, it does not appear to significantly influence Apgar scores. Other factors may be more relevant in determining neonatal conditions at birth.

No significant differences were found between the mothers with and without antepartum anemia regarding the length of neonatal hospitalization (t = -0.541, *P* = 0.589). Although infants born to mothers without anemia had slightly shorter hospital stays on average, the difference was not statistically significant, suggesting that antepartum anemia did not substantially impact the duration of neonatal hospitalization.

### Correlations between prenatal care and antibiotic administration in newborns

**Figure 8:**
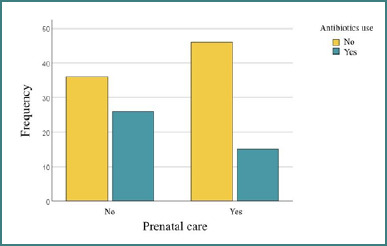
Correlations between prenatal care and antibiotic use in newborns

Other factors, such as neonatal infections, appear to have a greater influence on the length of hospitalization than antepartum anemia, which does not seem to play a major role in this context. There was a significant association between prenatal care and antibiotic administration in newborns (*P* = 0.041), suggesting that infants born to mothers who accessed prenatal care were less likely to receive antibiotics. These findings are illustrated in Figure 8. The administration of antibiotics may be associated with more severe cases or complications, leading to a longer hospitalization. There were significant differences between infants who received antibiotics and those who did not regarding the number of days of hospitalization (t = -4.693, *P* = 0.001).

## DISCUSSION

Prenatal visits represent a cornerstone of preventative healthcare, crucial in minimizing risks to maternal and fetal health. These visits facilitate the systematic monitoring of various physiological parameters, enabling the early detection of potential complications such as anemia, gestational diabetes, pre-eclampsia, and infections [2,3]. Timely intervention and appropriate treatment of these conditions can significantly mitigate adverse outcomes, reducing the need for extended hospital stays following birth [1,13]. The benefits of prenatal care extend beyond the purely clinical domain, encompassing improvements in psychological well-being during pregnancy and postpartum. By providing education, counseling, and emotional support, prenatal care empowers women to make informed decisions about their health and the health of their infants, fostering a sense of control and reducing anxiety [5,10].

Although the proportions were relatively similar, a marginal majority of patients in this cohort did not receive prenatal care services. A statistically significant correlation was observed between advanced maternal age and prenatal care utilization. This may be attributed to a heightened awareness of potential pregnancy-related complications among older individuals, leading to increased engagement with healthcare providers or to improved access to healthcare resources for this demographic. Primiparous women (first-time mothers) demonstrated a greater propensity to enroll in antenatal care programs than multiparous women (those with prior births). These findings suggest a complex interplay between maternal age, parity, and access to or engagement with prenatal care services, warranting further investigation to elucidate the underlying mechanisms and potential barriers.

Antepartum anemia was prevalent within a substantial segment of the studied population. Implementing prenatal care interventions was demonstrably associated with a reduced incidence of antepartum anemia, underscoring the critical role of systematic prenatal monitoring in mitigating associated risks. Notably, women who actively participated in prenatal care programs predominantly exhibited mild forms of anemia, with no instances of severe anemia documented within this subgroup. Conversely, the cohort of women who did not receive prenatal care displayed a more heterogeneous distribution of anemia severity, including the presence of severe anemia cases. This disparity highlights the protective effect of prenatal care in preventing the progression of anemia during pregnancy.

A statistically significant difference was identified in postpartum hemoglobin levels between patients who availed themselves of prenatal care services and those who did not engage in such medical consultations. Specifically, patients who underwent prenatal monitoring had significantly elevated postpartum Hb values compared to their counterparts. This observation underscores the beneficial impact of prenatal care on maternal hematological status following parturition.

Regarding neonatal outcomes, a significant association was observed between the provision of prenatal care and a reduced duration of hospital stay for newborns. While it is acknowledged that various factors, such as neonatal infections, can influence the length of hospitalization for newborns, and while antepartum anemia does not appear to be a primary determinant, a notable correlation exists between the absence of prenatal care, the administration of antibiotics to newborns, and the overall duration of their hospital stay. This suggests that inadequate prenatal care may contribute to adverse neonatal outcomes, potentially increasing the risk of infections and prolonged hospitalization. Further research is warranted to fully elucidate the complex interplay between prenatal care, neonatal health, and hospital length of stay.

The limitations of this study include a relatively small sample size and the possibility of confounding variables that can influence the results and make it difficult to establish causality.

## CONCLUSION

Proper prenatal care plays a vital role in reducing maternal and fetal morbidity and mortality. By proactively addressing potential health issues, optimizing clinical and psychological outcomes, and promoting healthy behaviors, prenatal care contributes significantly to the overall well-being of pregnant women and their offspring. The evidence overwhelmingly supports the implementation of comprehensive prenatal care programs as a critical strategy for improving reproductive health outcomes and reducing healthcare disparities. Further research is needed to identify and address barriers to prenatal care access, ensuring that all women, regardless of socioeconomic status or geographic location, can benefit from these essential services. Investment in prenatal care represents a cost-effective approach to promoting healthier pregnancies, healthier babies, and healthier communities.

## Data Availability

The raw data supporting the conclusions of this article will be made available by the authors on request.
